# The role of HLA genotypes in understanding the pathogenesis of severe COVID-19

**DOI:** 10.1186/s43042-023-00392-3

**Published:** 2023-01-26

**Authors:** Fatemeh Arab, Samaneh Mollazadeh, Farnaz Ghayourbabaei, Meysam Moghbeli, Ehsan Saburi

**Affiliations:** 1 Medical Genetics and Molecular Medicine Department, School of Medicine, Mashhad University of Medical Sciences, Mashhad, Iran; 2 Natural Products and Medicinal Plants Research Center, North Khorasan University of Medical Sciences, Bojnurd, Iran; 3 Department of Biology, Faculty of Sciences, University of Ferdowsi, Mashhad, Iran

**Keywords:** HLA, COVID-19, Prognosis

## Abstract

The coronavirus disease 2019 (COVID-19) pandemic has caused human tragedy through the global spread of the viral pathogen SARS-CoV-2. Although the underlying factors for the severity of COVID-19 in different people are still unknown, several gene variants can be used as predictors of disease severity, particularly variations in viral receptor genes such as angiotensin-converting enzyme 2 (ACE2) or major histocompatibility complex (MHC) genes. The reaction of the immune system, as the most important defense strategy in the case of viruses, plays a decisive role. The innate immune system is important both as a primary line of defense and as a trigger of the acquired immune response. The HLA-mediated acquired immune response is linked to the acquired immune system. In various diseases, it has been shown that genetic alterations in components of the immune system can play a crucial role in how the body responds to pathogens, especially viruses. One of the most important host genetic factors is the human leukocyte antigen (HLA) profile, which includes HLA classes I and II and may be symbolic of the diversity of immune response and genetic predisposition in disease progression. COVID-19 will have direct contact with the acquired immune system as an intracellular pathogen after exposure to the proteasome and its components through class I HLA. Therefore, it is assumed that in different genotypes of the HLA-I class, an undesirable supply causes an insufficient activation of the immune system. Insufficient binding of antigen delivered by class I HLA to host lymphocytes results in uncertain identification and insufficient activation of the acquired immune system. The absence of secretion of immune cytokines such as interferons, which play an important role in controlling viral infection in the early stages, is a complication of this event. Understanding the allelic diversity of HLA in people infected with coronavirus compared with uninfected people of one race not only allows identification of people with HLA susceptible to COVID-19 but also provides better insight into the behavior of the virus, which helps to take effective preventive and curative measures earlier.

## Introduction

A new coronavirus known as severe acute respiratory syndrome coronavirus 2 (SARS-CoV-2) was first reported in Wuhan, China, in December 2019. The rapid spread of this viral pathogen around the world has resulted in 619 million confirmed cases of infection and more than 6,537,636 deaths by October 2022 [1–3]. This virus is similar to a number of previous beta coronaviruses such as SARS and middle east respiratory syndrome (MERS), which can cause lower respiratory tract infections and even death [4, 5]. Of note, the mortality rate for the new coronaviruses is 3.78%, which is lower than two previous pandemics, SARS-CoV (15%) and MERS (37%) [6, 7]. Reportedly, the severity of infection of SARS-COV-2 varies from mild symptoms [8] to severe illness and even death. The incubation period (the time between exposure and the onset of symptoms) can be up to two weeks [9]. Fever, fatigue, and dry cough are the most common symptoms of COVID-19. Various other symptoms may also occur, such as pain and bruising, stuffy nose, runny nose, sore throat, or diarrhea. To relieve the symptoms, infected people should receive supportive treatment [6]. Although the world is fighting the epidemic COVID-19 by implementing quarantine measures and protocols as well as vaccinations [10], in reality, these efforts have been thwarted by many problems such as the confiscation of vaccines and medical supplies [11, 12].

### Host immunity against SARS-Cov-2

Viral infections can be effectively controlled by appropriate activation of cytotoxic T cells in response to antigen-presenting cells [13]. In this context, cluster of differentiation 4 (CD4) T cells (TCD4) play an important role in host immunity against SARS and MERS by stimulating the production of virus-specific antibodies via B cells. Moreover, cytotoxic TCD8 cells can kill infected cells by recognizing MHC on the cells [14], highlighting the important role of T cells in controlling the pathogenesis of these viral infections. On the other hand, T helper cells control infection by producing inflammatory cytokines [15]. However, the abnormal release of cytokines such as some interleukins (IL 6, IL 1, IL 2, IL 10), tumor necrosis factor-alpha (TNFα), and interferon-gamma (IFN-γ) causes an uncontrolled response that leads to the destruction of lung tissue and even death [16–19], phenomenon known as the cytokine storm.

A cytokine storm describes how the immune system contributes to an uncontrolled and widespread inflammatory response [20]. A cytokine storm is a major contributor to a more severe clinical course, as higher levels of CXCL10, CCL2, and TNFα were found in patients with COVID-19 who required admission to the ICU than in those who did not [21]. The immune system's "attack" on the body immediately follows the cytokine storm, and in the most severe cases of COVID-19 infection, the result is death [4]. It has been reported that among the interleukins, IL-6 plays a more important role in the cytokine storm caused by coronavirus due to its involvement in the regulation of the acute phase response [22]. However, much less is known about the biochemical and clinical implications of this immune system hyperactivity.

The presentation of viral fragments on the surface of host cells could be via MHC molecules, often referred to as human leukocyte antigen (HLA). The MHC, located on chromosome 6, plays an important role in the development of the immune response to protein antigens [2]. MHCs are classified into three classes based on their tissue distribution and function. Epidemiological studies have shown an association between various diseases and certain HLA alleles, including those caused by ribonucleic acids (RNA) viruses, such as SARS, influenza, human immunodeficiency virus (HIV), hepatitis C, rabies, and other [23]. Compared with HLA class II, HLA class I plays a crucial role in viral infection by presenting viral antigens to CD8^+^ T cells on the surface of infected cells, followed by recognition and destruction of the cells [24]. The likely effect of T cells in SARS-CoV-2 infection could be illustrated by the response of 40–60% of T cells in unexposed individuals to viral proteins due to a cross-immune reaction with other coronaviruses in previous colds [25].

### Pathogenesis of SARS-CoV-2

Understanding the susceptibility or resistance to a particular disease associated with the presence of specific alleles can be useful in drug manufacturing and development and in identifying at-risk populations [26]. In this regard, COVID-19 can be prevented by lifelong immunological memory using a vaccine based on natural protective immunity to SARS-CoV-2 infection [27, 28]. Therefore, it is important to find the reasons for the different clinical responses of infected individuals. In this context, reference can be made to polymorphisms in various genes, especially in viral receptor genes (ACE2) or genes involved in the diversity of immune responses (such as MHCs). Angiotensin-converting enzyme 2 (ACE2) is a protein with multiple functions, including catalytic, amino acid transporter, and viral receptor [29]. The host receptor of SARS-CoV-2, ACE2, binds to cell membranes and acts as a transporter of the new virus [14]. Coronavirus entry is mediated by the spike S glycoprotein [30]. Following viral binding and membrane fusion, ACE2 is internalized, and its activity is downregulated on the target cell surface, leading to COVID-19 infection [1].

MHC class I/II may be a symbol of the diversity of a person's immune response and genetic predisposition to disease progression and immunity [31]. Detection of differences in HLA response to SARS-CoV-2 peptides in infected patients could be a potential factor for developing a personalized treatment based on individual risk. HLA polymorphism in different populations could have an impact on the susceptibility and severity of COVID-19. In this context, a common specific allele may be more prevalent in a given population than others. In contrast, various HLA alleles in different populations might have similar binding sites for viral peptides [32, 33].

### Antigen presentation in SARS-CoV-2 infection

**Fig. 1:**
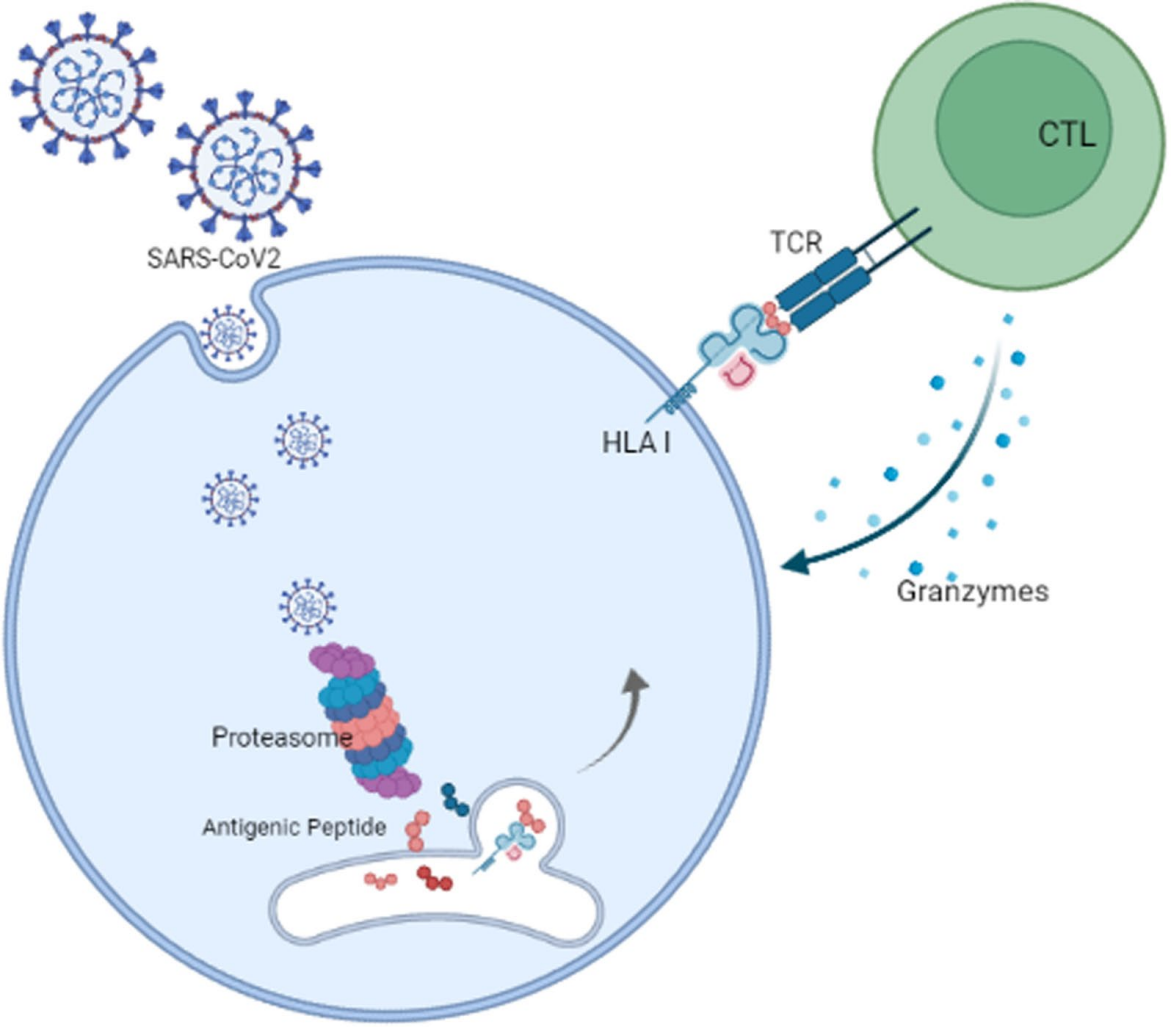
SARS-CoV-2 antigen presentation pathway through MHC-I molecules. Cells infected with SARS-CoV-2 produce various isoforms of endoplasmic reticulum aminopeptidase 2 (ERAP2) genes dimerized with either ERAP2-wild type (wt) or ERAP1-wt which can be presented on cells and recognized by specific CD8 + cytotoxic T lymphocytes (CTL). ER: endoplasmic reticulum; TAP: transporter associated with antigen processing [36]. Created with BioRender.com

Specifically, MHC-I presents viral antigens via the direct involvement of endoplasmic reticulum aminopeptidase (ERAP) isoforms, ERAP1 and ERAP2 proteins, which in turn can contribute to the recognition of the infected cell by CD8 + cytotoxic T lymphocyte (CTL) clones and trigger a protective immune response [34]. As shown in Fig. 1, during replication of SARS-CoV-2 in host cells, viral antigens are processed by host proteasomes, and the resulting peptides are transported to the endoplasmic reticulum (ER) by the molecular transporter associated with antigen processing (TAP) [35]. In the endoplasmic reticulum, viral peptides are influenced by ERAP1 and ERAP2 to be presented in the clefts of MHC class I molecules. Finally, MHC I enables the monitoring of ongoing infections by CD8 + T cells [36].

## Discussion

### HLA genotypes and SARS-CoV-2

As shown in Table 1, many studies have been performed on the HLA types involved in the susceptibility or severity of COVID-19. In describing these studies, we first discuss the significant relationship between viral infections with a similar pathogenicity mechanism as SARS-CoV-2 and the HLA system. Then, we will evaluate SARS-CoV-2 in this regard. For example, Nguyen et al. identified HLA-B*15:03 and found that individuals with this allele were more able to deliver viral peptides to the cell surface. In contrast, they predicted that the HLA-B*46:01 allele was the least able to bind to viral peptides, suggesting that individuals with this allele may have a weaker immune response and more severe symptoms [26]. In HIV-1 infection, which has a similar pathogenic mechanism to COVID-19, the presence of HLA-A*02:05 results in relative resistance to the disease. However, in the Thai population, some HLA alleles, including HLA-A*02:07 and HLA-B*51, have been associated with increased disease severity [37, 38].

**Table 1:** Types of HLA that are involved in the susceptibility or severity of COVID-19

Authors (ref)	Year	Locality	People/ Database	HLA variants	Trait
Abdelhafiz et al. [56]	2022	Egypt	69 people with COVID-19/ mild/ moderate/ severe	HLA-B*15	Protective
Yung et al. [47]	2021	Hong Kong	190 people with COVID-19/ 3892 healthy controls	HLA-B*22	Susceptibility
Shkurnikov et al. [57]	2021	Moscow, Russia	111 people with COVID-19/ 428 healthy controls	HLA-A*02:01 HLA-A*03:01 HLA-A*01:01	Protective Protective Susceptibility
Anzurez et al. [58]	2021	Japan	30 mild, 75 moderate, 51 Severe and 22 Critical COVID-19	HLA- DRB1*09:01	Susceptibility
Weiner et al. [59]	2021	4 Countries (Germany, Spain, Switzerland and the United States)	435 people with COVID-19	HLA-C*04:01	Susceptibility
Langton et al. [60]	2021	North East of England	147 people with COVID-19/ 8514 healthy controls/ IRAS project 283,409; REC reference: 20/YH/0184	HLA-DRB1*04:01 HLA-DRB1*01:01	Protective Protective
Romero Lopez et al. [61]	2021	28 states of Mexico	TepiTool server from the IEDB Analysis Resource database/ Total: 71,099	HLA-DRB1*01	Susceptibility
L Warren et al. [62]	2021	New York cohort	100 people with COVID-19/ 26 healthy controls	HLA-DPA1*02:02 HLA-C*04:01 HLA- A*11:01	Susceptibility Severity severity
M A Naemi et al. [33]	2021	South Asia (Bangladeshis, Indians, and Pakistanis)	64 mild, 31 severe and 20 fatal COVID-19	HLA-B*51 HLA-B*35	Susceptibility Protective
Sakuraba et al. [54]	2020	74 countries	The Allele Frequency Net Database and worldometer.info	HLA-C*05	Susceptibility
Novelli et al. [50]	2020	Italy	99 severe or extremely severe COVID-19/ 1017 healthy controls	HLA-B*27:07	Susceptibility
Wang et al. [63]	2020	China/ Han people	82 people with COVID-19/3548 healthy controls	HLA-C*07:29 HLA-B*15:27	Susceptibility Susceptibility
Lorente et al. [64]	2020	Spain/ Canary Islands	72 severe COVID-19/ 3886 healthy controls	HLA-B*39 HLA-C*16	Susceptibility Susceptibility
Correale et al. [65]	2020	Italy (different regions)	Italian Bone Marrow Donors Registry (IBMDR) high-definition-analysis database	HLA-B*44 HLA-C*01	Susceptibility Susceptibility
Pisanti et al. [49]	2020	Italy	IBMDR high-definition-analysis database	HLA-A*01:01 HLA-B*08:01 HLA-C*07:01 HLA-DRB1*030:1	Susceptibility Susceptibility Susceptibility Susceptibility
Warren et al. [1]	2020	China/ Wuhan	5 patients with COVID-19	HLA-A*24:02	Susceptibility
Tomita et al. [37]	2020	19 Countries	(Allele Frequency Net Database)/ An in silico analysis	HLA-A*02:01 HLA-A*24:02 HLA-A*11:01	Susceptibility Protective protective
Toyoshima et al. [39]	2020	28 countries	12,343 SARS-CoV-2 genome sequences isolated from patients/the reference SARS-CoV-2 sequence	HLA-A*11:01	protective
Lorente et al. [64]	2020	Spain/ Canary Islands	72 severe COVID-19/ 3886 healthy controls	HLA-A*32	protective
Iturrieta et al. [28]	2020	Spain	5 mild, 20 moderate and 20 severe COVID-19	HLA-B*15:03	protective
Pisanti et al. [49]	2020	Italy	IBMDR high-definition-analysis database	HLA-A*02:01 HLA-B*18:01 HLA-C*07:01	Protective protective protective
Littera et al. [66]	2020	Sardinian	182 SARS-CoV-2 patients/619 healthy controls	HLA-A*02:05 HLA-B*58:01 HLA-C*07:01	Protective Protective protective

Notably, HLA-Cw1502, DR0301, HLA-Cw*1502, DRB1*0301, HLA-B*15:02, A*02:06, A*68:01, A*02:22, and A*24:03 confer resistance to SARS-CoV-1 infection [39, 40]. It has also been reported that the MHC class I haplotype (HLA-B*-4601, HLA-B*-0703, HLA-Cw* 0801, HLA-A*11:01, B*51:01, C* 14:02) and the MHC class II haplotype, HLA-DRB1*1202, are associated with increased susceptibility to SARS-CoV-1 [40–42]. Because the homology and pathogenicity mechanism of SARS-Co-V are very similar to COVID-19, the HLAs likely mentioned in various studies, such as HLA-A*02:01, HLA-A*02:06, HLA-A*24:02, HLA-B*15:03, HLA-B*44, C*01, HLA-A*25, and HLA-B*08, also play a vital role in susceptibility or resistance to COVID-19 [39, 43, 44].

To better understand the relationship between HLA alleles and SARS-CoV-2 outcomes, we will describe additional studies in this section. The first study examined HLA-A*24:02 in a small group of the Wuhan population. Warren et al. found this allele in four of five individuals and described it as an allele associated with susceptibility to SARS-Cov-2 [45]. After the publication of the current article, the results of the study by Tomita et al. showed that individuals carrying the alleles HLA-A*24:02 and HLA-A*11:01 have a relatively higher ability to present SARS-CoV-2 antigens than individuals carrying HLA-A*02:01. In other words, they mentioned HLA-A*02:01 as a susceptible allele for COVID-19 [46]. In another study, 190 patients infected with SARS-CoV-2 in Hong Kong were found to have an association between serotype HLA-B*22 and an increased risk of COVID-19 [47].

The association between mortality rate and frequency of HLA allele haplotypes was found in 28 countries. In addition, the HLA-A*11:01 allele was found to be associated with a lower mortality rate [48]. In addition, three studies have been conducted in different regions of Italy to investigate the HLA allele haplotype and the incidence and mortality of COVID-19. The first study found that HLA-B*44 and HLA-C*01 allele groups were associated with an increased incidence of COVID-19 [43]. In the second study, the authors found that HLA-A*01:01, B*08:01, C*07:01, and DRB1*030:1 were associated with increased incidence and mortality of COVID-19, whereas the HLA-A*02:01-B*18:01-C*07:01-BRB1*11:04 haplotype was associated with lower incidence and mortality [49]. In the third study, 99 Italian patients infected with the severe form of COVID-19 were compared with a control group of 1017 uninfected individuals. The results showed that the following HLA alleles were susceptible to SARS-CoV-2: HLA-B*27:07 from MHC class I and HLA-DRB1*15:01 and HLA-DQB1*06:02 alleles from MHC class II [50].

In another case–control study in China, two HLA-I alleles at high risk for SARS-CoV-2 were identified: HLA-C*07:29 and HLA-B*15:27 [44]. A study examining a small number of mild, moderate, and severe forms of COVID-19 patients in Spain also found that the number of SARS-CoV-2 peptides bound to HLA molecules was negatively associated with disease severity. Theoretically, the higher affinity of HLA-I to bind to SARS-CoV-2 peptides was associated with the less severe forms of the disease [51].

Accordingly, the genome-wide association study (GWAS), involving 835 patients with severe forms of COVID-19 from Italy and 775 patients from Spain, did not identify any HLA alleles associated with the development of infection or disease severity. Similar to the previous study, the high number of SARS-COV-2 peptides binding in the cleft of HLA-I molecules was found to be associated with the less severe forms of the disease [52].

It should be noted that information from studies with a large statistical population does not always show a significant association between HLA and COVID-19. For example, in a study of 3886 healthy controls and 72 COVID-19 patients by Lorente et al. the HLAA*32 allele was found to be a protective allele, whereas the frequency of the HLA-C*16 and HLA-B*39 alleles was higher in infected individuals; however, the results were not statistically significant [53].

Sakuraba et al. examined the frequency of HLA class I alleles in 74 countries around the world using the Allele Frequency Net Database and the website www.worldometer.info. The results supported an association between the HLA-C*05 allele and higher mortality after developing COVID-19 [54]. Evaluation of HLA for SARS-CoV-2 severity in the Sardinian population also showed a protective effect of the following HLA haplotypes against SARS-CoV-2: HLA-A*02:05, B*58:01, C*07:01, and DRB1*03:01 [55].

Some studies have also examined the relationship between COVID-19 and HLA in patients with certain diseases, such as cancer. For example, no significant allelic relationship was found in the HLA genotype of lung cancer patients with and without COVID-19 [24].

SARS-CoV-2 mutations not only influence the COVID-19 course of the pandemic but also have a major impact on T-cell immunity, depending on the HLA supertype. Specifically, certain types of mutations in the viral genome trigger a variety of CD8 + T cell targets. Mutational biases also affect epitope presentation in a manner that depends on the HLA supertype. An important point regarding the dependence on HLA supertype in infection with mutant variants of SARS-CoV-2 is the differential modulation of T cell responses in different populations [27]. Based on the genetic landscapes of different populations, a way to predict infections and evaluate the efficacy of vaccines could be paved.

## Conclusion

Although some studies have identified a specific HLA allele or allele group significantly associated with COVID-19 severity, the resulting data appear to be controversial. In addition, in the largest study examining more than 1500 patients with severe forms of COVID-19, no allelic association was found. Therefore, further studies should be performed to clarify the relationship between HLA alleles and COVID-19 development. Because of the diversity of HLAs and their various ethnic-genetic and geographic distributions [67–70], it is essential to study the susceptible and protective HLA alleles against SARS-CoV-2 in each race to take effective preventive and curative measures.

## Data Availability

Not applicable.

## Abbreviations

ACE2: Angiotensin- converting enzyme 2
COVID-19: Coronavirus disease 2019
CD4: Cluster of differentiation 4
CTL: Cytotoxic T lymphocyte
ERAP: Endoplasmic reticulum aminopeptidase
ER: Endoplasmic reticulum
GWAS: Genome-wide association study
HLA: Human leukocyte antigen
HIV: Human immunodeficiency virus
IL: Interleukins
IFN-γ: Interferon-gamma
MHC: Major histocompatibility complex
MERS: Middle east respiratory syndrome
RNA: Ribonucleic acids
SARS-CoV-2: Severe acute respiratory syndrome coronavirus 2
TNFα: Tumor necrosis factor-alpha
TAP: Transporter associated with antigen processing
